# Clinical Features and Outcomes of Primary Colorectal Diffuse Large B‐Cell Lymphoma: A Multicenter Retrospective Study

**DOI:** 10.1002/cam4.71313

**Published:** 2025-10-21

**Authors:** Yong‐Pyo Lee, Myung‐Won Lee, Seonggyu Byeon

**Affiliations:** ^1^ Division of Hematology‐Oncology, Department of Internal Medicine Chungbuk National University Hospital, Chungbuk National University College of Medicine Cheongju Republic of Korea; ^2^ Division of Hematology/Oncology, Department of Internal Medicine Chungnam National University Hospital, Chungnam National University College of Medicine Daejeon Republic of Korea; ^3^ Catholic Hematology Hospital, Seoul St. Mary's Hospital, College of Medicine The Catholic University of Korea Seoul Republic of Korea

**Keywords:** primary colon diffuse large B‐cell lymphoma, primary gastrointestinal diffuse large B‐cell lymphoma, retrospective analysis

## Abstract

**Background:**

Primary gastrointestinal (GI) diffuse large B‐cell lymphoma (DLBCL) is a rare malignancy. Given its rarity, the nature of the disease, particularly those originating in the colorectum, remains poorly defined.**Aims:** This multicenter retrospective study analyzed the clinical characteristics and treatment outcomes of primary GI DLBCL, with a focus on colorectal cases.

**Materials & Methods:**

A total of 104 cases of primary GI DLBCL were retrospectively collected from three institutions (2010–2024) and classified into three groups based on the lymphoma's origin.

**Results:**

Among 104 patients, 57.7% had gastric, 18.3% small bowel, and 24.0% colorectal DLBCL. Approximately 60% presented with limited‐stage disease (Stage I–II). All patients received front‐line R‐CHOP (rituximab, cyclophosphamide, doxorubicin, vincristine, and prednisone), achieving a complete remission (CR) rate of 81.0%. The estimated 3‐year overall survival (OS) and progression‐free survival (PFS) were 91.7% and 91.9%, respectively. Outcomes varied by disease origin, with gastric DLBCL showing the most favorable prognosis and small bowel the poorest (3‐year OS 93.9% vs. 69.3%).

**Discussion:**

In the colorectal subgroup (*n* = 25), 84.0% had disease in the ascending colon, and 70.0% had limited‐stage disease. Obstructive symptoms were the most common initial presentation. The CR rate after R‐CHOP was 80.0%, with estimated 3‐year OS and PFS of 86.7% and 72.3%, respectively. While primary tumor resection improved local disease control, it did not confer an OS benefit. During follow‐up, 13.5% of patients experienced relapse, most frequently more than 12 months after achieving CR. Relapsed or refractory primary GI DLBCL demonstrated better outcomes than conventional relapsed nodal DLBCL.

**Conclusion:**

These findings confirm the efficacy of front‐line R‐CHOP in primary GI DLBCL and suggest that primary tumor resection may be a useful component of treatment for localized primary colorectal DLBCL.

## 1 Introduction

Although primary gastrointestinal (GI) lymphoma represents a relatively rare subset of all GI malignancies [1], the GI tract remains the most frequently involved site of extra‐nodal lymphomas, reflecting its unique immunological environment and extensive lymphoid tissue distribution throughout the mucosa‐associated lymphoid tissue system [2, 3]. Among these sites, the stomach is the most frequently affected, with diffuse large B‐cell lymphoma (DLBCL) being the predominant subtype [4]. Although data on primary gastric DLBCL are relatively abundant, information on primary intestinal DLBCL remains scarce, with only a few case reports and small sample sizes, which hinder a comprehensive understanding of its clinical features and treatment outcomes.

While primary intestinal DLBCL exhibits distinct clinical and genetic characteristics [5], treatment strategies and outcome predictions have largely been extrapolated from those of conventional nodal DLBCL In contrast to primary gastric DLBCL, where surgical intervention is infrequently used [6], standard treatment for primary intestinal DLBCL typically involves a combination of surgical resection and immunochemotherapy (rituximab, cyclophosphamide, doxorubicin, vincristine, and prednisone; R‐CHOP) [7, 8]. However, the role and extent of surgery should to be re‐evaluated, especially now that rituximab is routinely used. This is particularly important due to the potential for surgery‐related morbidity and its impact on quality of life. Furthermore, the role and indications of adjuvant treatment, such as radiation therapy (RT) or autologous stem cell transplantation (ASCT), in primary intestinal DLBCL remain undefined, despite their frequent application in other GI malignancies.

This retrospective study reports the clinical characteristics and treatment outcomes of primary GI DLBCL based on its origin, with a particular focus on the rare entity of primary colorectal DLBCL. Real‐world data were analyzed to identify disease features and explore potential prognostic factors.

## 2 Methods

### 2.1 Study Analysis and Patient Collection

**FIGURE 1:**
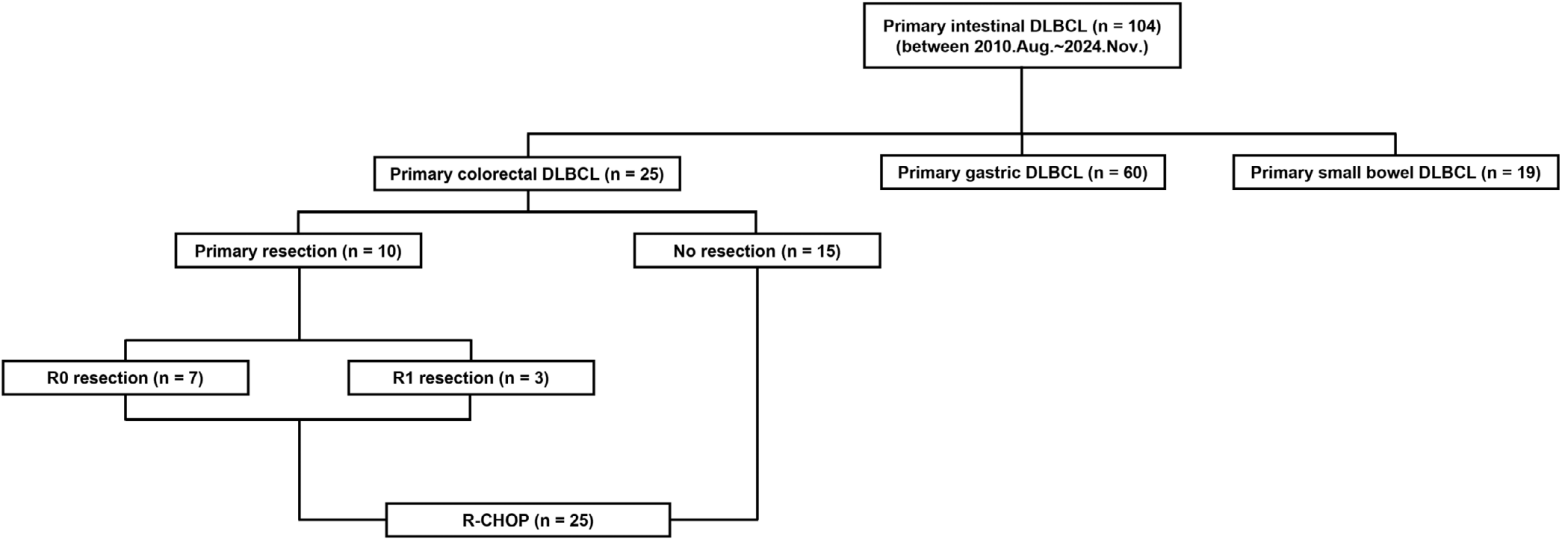
Flow diagram of the study population.

A total of 104 cases of primary GI DLBCL was retrospectively collected from three institutions between 2010 and 2024. The patients were classified into three groups based on the origin of lymphoma: primary gastric DLBCL (*n* = 60, 57.7%), primary small bowel DLBCL (*n* = 19, 18.3%), and primary colorectal DLBCL (*n* = 25, 24.0%) (Figure 1). Each GI lymphoma was defined based on its site of origin as follows: primary gastric DLBCL was defined as lymphoma originating between the esophagogastric junction and pylorus; primary small bowel DLBCL included cases arising from the first portion of the duodenum to the terminal ileum; and primary colorectal DLBCL encompassed lesions from the cecum to the rectum. In cases involving both the ileum and cecum, classification was based on the predominantly affected site as determined on radiologic or endoscopic findings. Since the optimal method for distinguishing primary GI DLBCL from conventional nodal DLBCL secondarily involving the GI tract is yet to be determined, patients with predominant GI lesions were classified with primary GI DLBCL when the disease predominantly involved the GI tract based on imaging assessments, in accordance with previously described criteria [9].

Pathologic diagnosis was performed by experts in lymphoid malignancy pathology at each institution using immunohistochemistry results according to the World Health Organization diagnostic criteria [10]. The cell of origin of lymphoma was determined using the Hans algorithm [11]. Clinical information from patients, including age, sex, Eastern Cooperative Oncology Group performance status, disease stage, extra‐nodal site involvement, complete blood count, lactate dehydrogenase (LDH), and beta‐2 microglobulin levels was obtained to evaluate clinical prognosis. The revised international prognostic index (R‐IPI) was estimated for each patient at the time of diagnosis. In particular, the disease stage was assessed using the Lugano staging system for GI lymphoma [12]. At diagnosis, baseline imaging studies including neck, chest, and abdominopelvic computed tomography (CT) scans as well as ^18^F‐fluorodeoxyglucose positron‐emission tomography/CT (PET‐CT) scans were conducted. All patients, except those with primary small bowel DLBCL, also underwent esophagogastroduodenoscopy (EGD) and/or colonoscopy. Bone marrow (BM) examination was performed in most patients at the time of diagnosis, although it was omitted in a small number of cases with advanced‐stage disease due to clinical considerations.

The institutional review boards of the participating hospitals approved this study, and written informed consent was waived due to the retrospective design based on medical record review.

### 2.2 Primary Treatment

Information on primary treatment was collected including surgical resection of the primary lymphoma lesion, type of immunochemotherapy regimen administered, and use of consolidative treatments such as RT or ASCT. The decision to perform surgical resection was made in accordance with institutional policies, with the extent of resection evaluated as R0 (absence of microscopic residual disease at the resection margin) or R1 (presence of microscopic residual disease at the resection margin). All patients, regardless of surgery, were recommended to receive four to six cycles of immunochemotherapy. Treatment response was assessed after completing the planned treatment according to the Lugano classification for lymphoma using CT and PET‐CT scans in patients with measurable nodal or extranodal lesions [13]. A limited number of patients received consolidative treatment, with the choice of treatment determined at the discretion of the attending physician. Following completion of primary treatment, patients were monitored for disease relapse with laboratory tests and CT scans every 3 to 6 months.

### 2.3 Statistical Analysis

Descriptive statistics included percentages and medians, and the intergroup comparisons of categorical variables were performed using the χ^2^ or Fisher's exact test. The Kaplan–Meier method was used to determine progression‐free survival (PFS) and overall survival (OS). PFS was estimated from diagnosis to disease progression or death from any cause. OS time was assessed from diagnosis to death or the last follow‐up date. In addition, post‐progression OS (OS2) was defined as the time from disease progression following front‐line R‐CHOP therapy to the date of death from any cause and was censored at the date of the last available follow‐up. To elucidate factors associated with the survival of primary GI DLBCL, we performed univariate and multivariate analyses using the Cox proportional hazards model. All data were analyzed using the Statistical Package for Social Sciences software, version 24.0 (IBM Corp., Armonk, NY, USA).

## 3 Results

### 3.1 Characteristics of Patients With Primary GI DLBCL

Baseline characteristics at the time of diagnosis are shown in Table 1. The median patient age was 64 years (23–89 years). Approximately 60% of patients were aged 60 years or older, with most maintaining good performance status. Lymphoma was confined to the GI tract or adjacent lymph nodes (Stage I and II‐1) in 63% of cases, and disseminated extra‐nodal involvement or supra‐diaphragmatic lymph node involvement (Stage IV) was observed in about a quarter of patients. Consequently, two‐thirds of the patients had a favorable R‐IPI score. The majority of patients had non‐germinal center B‐cell type DLBCL. In addition, patient characteristics were analyzed and compared based on lymphoma origin; primary gastric DLBCL demonstrated more favorable features, whereas primary small bowel and primary colorectal DLBCL showed a higher incidence of poor R‐IPI scores due to the predominance of patients older than 60 years with poor performance status and advanced stage (Table 1).

**TABLE 1:** Comparison of baseline disease characteristics based on lymphoma origin.

Characteristics		Overall (*n* = 104)	Colorectal (*n* = 25)	Small bowel (*n* = 19)	Gastric (*n* = 60)	*p*
Age, years	Median (years), range	64 (23–89)	64 (23–82)	64 (47–84)	64 (34–89)	0.034
	< 60	43 (41.3%)	8 (32.0%)	4 (21.1%)	31 (51.7%)	
	≥ 60	61 (58.7%)	17 (68.0%)	15 (78.9%)	29 (48.3%)	
Sex	Male	55 (52.9%)	13 (52.0%)	9 (47.4%)	33 (55.0%)	0.840
	Female	49 (47.1%)	12 (48.0%)	10 (52.6%)	27 (45.0%)	
ECOG‐PS	0–1	65 (62.5%)	14 (56.0%)	6 (31.6%)	45 (75.0%)	0.002
	2–4	39 (37.5%)	11 (44.0%)	13 (68.4%)	15 (25.0%)	
Lugano stage	I	39 (37.5%)	6 (24.0%)	6 (31.6%)	27 (45.0%)	0.070
	II‐1	26 (25.0%)	11 (44.0%)	2 (10.5%)	13 (21.7%)	
	II‐2	3 (2.9%)	1 (4.0%)	0 (%)	2 (3.3%)	
	II‐E	12 (11.5%)	3 (12.0%)	2 (10.5%)	7 (11.7%)	
	IV	24 (23.1%)	4 (16.0%)	9 (47.4%)	11 (18.3%)	
Bone marrow involvement	Present	7 (6.7%)	0 (0.0%)	2 (10.5%)	5 (8.3%)	0.238
Initial B symptom	Present	12 (11.5%)	3 (12.0%)	5 (26.3%)	4 (6.6%)	0.065
R‐IPI	Very good (0)	18 (17.3%)	2 (8.0%)	3 (15.8%)	13 (21.7%)	0.082
	Good (1–2)	51 (49.0%)	12 (48.0%)	6 (31.6%)	33 (55.0%)	
	Poor (3–5)	35 (33.7%)	11 (44.0%)	10 (52.6%)	14 (23.3%)	
Serum LDH	Elevated	38 (36.5%)	9 (36.0%)	9 (47.4%)	20 (33.3%)	0.541
Cell of origin	Non‐GCB	63 (60.6%)	18 (72.0%)	12 (63.1%)	33 (55.0%)	0.429
	GCB	34 (32.7%)	7 (28.0%)	6 (31.6%)	21 (35.0%)	
	Not available	7 (6.7%)	0 (0.0%)	1 (5.3%)	6 (10.0%)	
Epstein–Barr Virus ISH	Positive	10 (9.6%)	3 (12.0%)	0 (0.0%)	7 (11.7%)	0.001

Abbreviations: ECOG‐PS, eastern cooperative oncology group performance status; GCB, germinal center B‐cell; ISH, in situ hybridization; LDH, lactate dehydrogenase; R‐IPI, revised international prognostic index.

### 3.2 Survival Outcomes of Patients With Primary GI DLBCL

**FIGURE 2:**
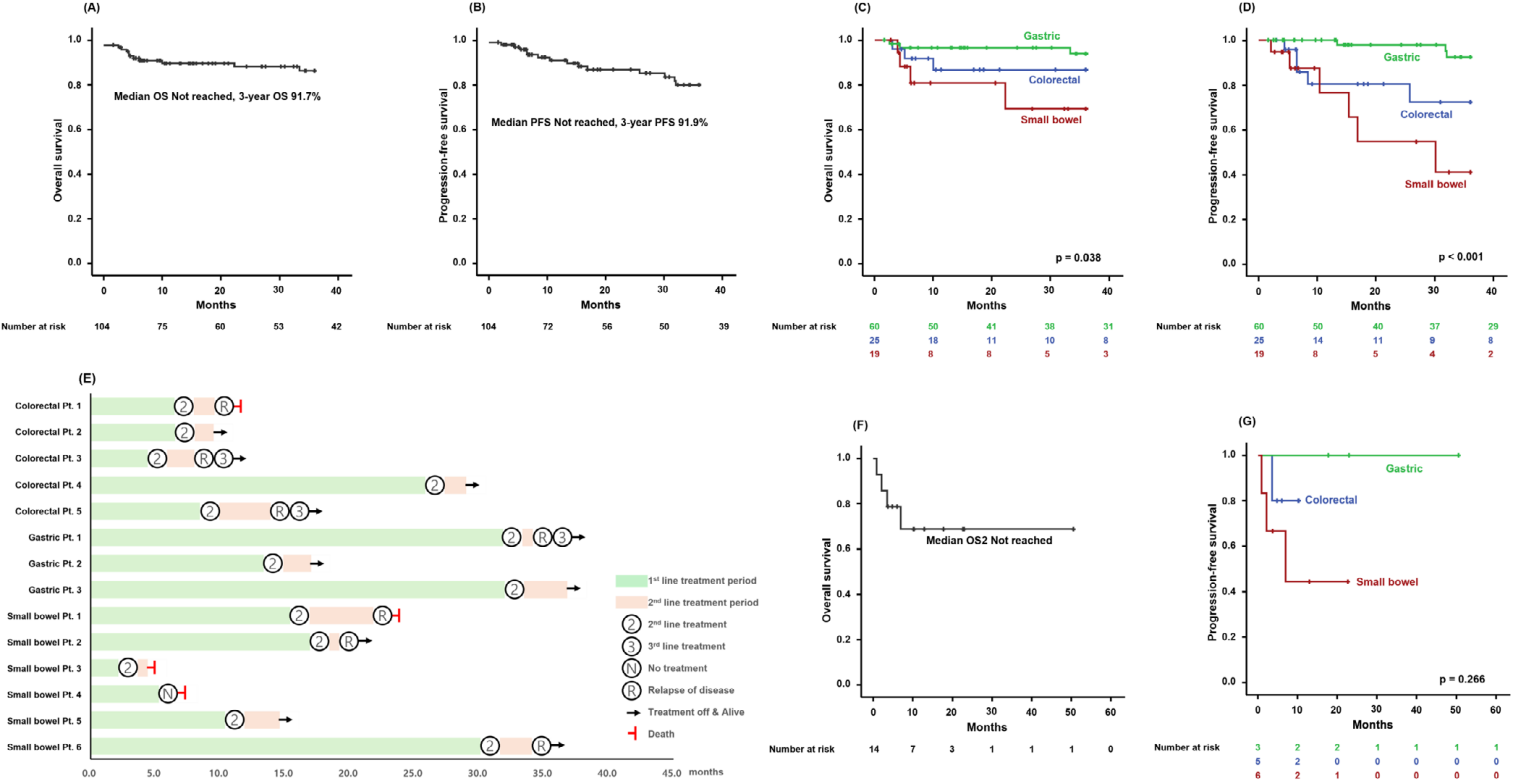
Kaplan–Meier curves of overall survival (OS) (A) and progression‐free survival (PFS) (B). Comparison of OS (C) and PFS (D) according to the origin of lymphoma. Swimmer plot for primary gastrointestinal diffuse large B‐cell lymphoma patients after relapse (E). Kaplan–Meier curves of post‐progression overall survival (OS2) (F). Comparison of OS2 according to the origin of lymphoma (G).

Of the 104 patients who uniformly received R‐CHOP as front‐line treatment, 81.0% (*n* = 85) achieved a complete remission (CR) at the end of treatment. Among five (4.8%) patients who were still undergoing front‐line therapy, four attained CR at the interim evaluation. Fourteen (13.5%) patients did not complete the planned treatment: four (28.6%) had primary refractory disease, defined as failure to achieve an objective response to front‐line therapy; three (21.4%) died from non‐lymphoma‐related causes, including two cases of sepsis and one case of pneumonia; and seven (50.0%) discontinued treatment due to treatment‐related toxicity. The median number of treatment cycles was 6 (range, 1–6). Throughout the study period, 10 death events were observed, of which four were attributed to refractory or relapsed lymphoma. With a median follow‐up duration of 36.1 months [95% confidence interval (CI), 25.34–46.85 months], OS and PFS did not reach the median values, and the estimated 3‐year OS and PFS for primary treatment were 91.7% and 91.9%, respectively (Figure 2A,B). Survival outcomes showed significant differences based on lymphoma origin, with particularly poor outcomes observed in primary small bowel DLBCL patients (Figure 2C,D). Following front‐line R‐CHOP therapy, five patients (4.8%) received consolidative treatment, with four undergoing RT and one receiving ASCT. Univariate and multivariate analyses revealed that elevated LDH was significantly associated with OS, while advanced stage and cell of origin were significant predictors of PFS (Table S1).

A total of 14 patients (13.5%) experienced disease relapse after primary treatment, with five (35.7%) originating from the colorectum, three (21.4%) from the stomach, and six (42.9%) from the small bowel (Figure 2E). Excluding four patients with primary refractory disease and one who discontinued treatment due to toxicity, nine (64.3%) had a relapse more than 12 months after achieving CR. The median time from CR to relapse was 30.1 months (range, 2.1–32.0 months). Among the five patients who received consolidative treatment, one (20.0%) experienced relapse and underwent RT. All but one relapsed patient received subsequent treatment. Among them, five patients (38.5%) achieved CR, seven (53.8%) had progressive disease, and one (7.7%) is currently receiving second‐line therapy. The median OS2 has yet to be reached, and the 1‐year OS2 rate was 66.8% (Figure 2F). Relapsed primary GI lymphoma showed a trend in survival differences based on its origin; however, the difference was not statistically significant (Figure 2G).

### 3.3 Subgroup Analysis of Primary Colorectal DLBCL

**FIGURE 3:**
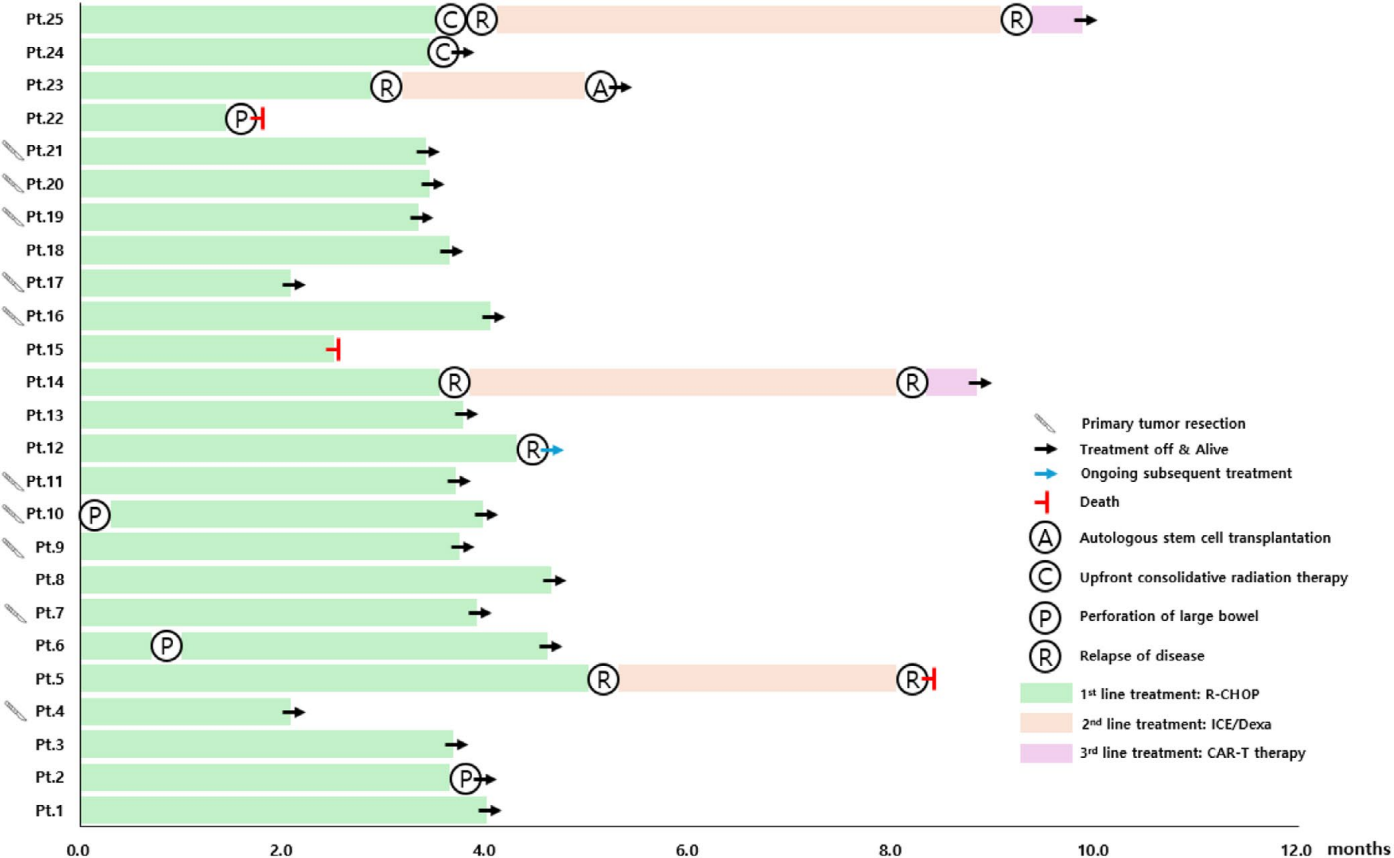
Swimmer plot for patients with primary colorectal diffuse large B‐cell lymphoma.

We performed a subgroup analysis focusing on 25 patients with primary colorectal DLBCL (Figure 3; Table 2). The majority (*n* = 21, 84.0%) had a primary lesion in the ascending colon, one (4.0%) had involvement in the transverse colon, two (8.0%) in the descending colon, and one (4.0%) in the sigmoid colon. Approximately 70% of patients (*n* = 17) presented with limited‐stage disease (Stage I or II‐1). Most patients (*n* = 22) presented with a protruded or ulcerofungating mass on colonoscopy, and a few exhibited diffuse colonic wall edema (*n* = 2) or intussusception (*n* = 1). Bulky disease of 7 cm or more was observed in nine patients (36.0%), and obstructive symptoms were the most common initial presentation in all patients (*n* = 15, 60.0%). Bleeding‐related symptoms were observed in seven patients (28.0%), and incidental detection occurred in only three cases (12.0%). A high proportion of patients with primary colorectal DLBCL had a poor R‐IPI (*n* = 11, 44.0%), likely due to older age and impaired performance status, despite the uncommon occurrence of extra‐nodal involvement, including BM.

**TABLE 2:** Baseline demographics and disease characteristics of primary colon DLBCL (*n* = 25).

Characteristics		*n* (%)
Age, years	Median (years), range	64 (23–82)
	< 60	8 (32.0%)
	≥ 60	17 (68.0%)
Sex	Male	13 (52.0%)
	Female	12 (48.0%)
ECOG‐PS	0–1	14 (56.0%)
	2–4	11 (44.0%)
Lugano stage	I	6 (24.0%)
	II‐1	11 (44.0%)
	II‐2	1 (4.0%)
	II‐E	3 (12.0%)
	IV	4 (16.0%)
Extra‐nodal involvement	≥ 2 sites	3 (12.0%)
Bone marrow involvement	Present	0 (0.0%)
Initial B symptom	Present	3 (12.0%)
Bulky (≥ 7 cm) mass	Present	9 (36.0%)
R‐IPI	Very good (0)	2 (8.0%)
	Good (1–2)	12 (48.0%)
	Poor (3–5)	11 (44.0%)
Anemia	Present	19 (76.0%)
Serum LDH	Elevated	9 (36.0%)
Cell of origin	Non‐GCB	18 (72.0%)
	GCB	7 (28.0%)
Epstein–Barr Virus ISH	Positive	3 (12.0%)
Primary lymphoma location	Ascending colon	21 (84.0%)
	Transverse colon	1 (4.0%)
	Descending colon	2 (8.0%)
	Below sigmoid colon	1 (4.0%)
Primary lymphoma resection	R0 resection	7 (28.0%)
	R1 resection	3 (12.0%)
	Not done	15 (60.0%)

Abbreviations: ECOG‐PS, eastern cooperative oncology group performance status; GCB, germinal center B‐cell; ISH, in situ hybridization; LDH, lactate dehydrogenase; R‐IPI, revised international prognostic index.

**FIGURE 4:**
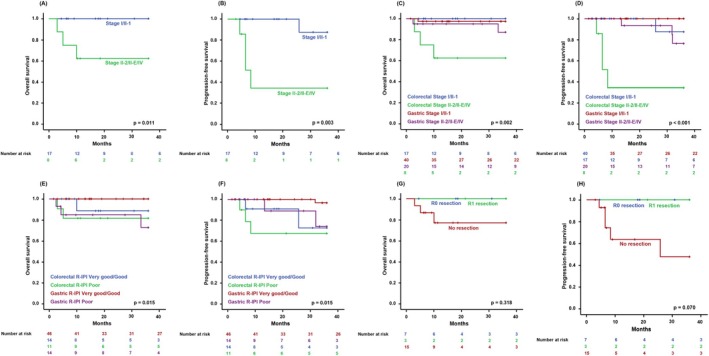
Comparison of overall survival (OS) (A) and progression‐free survival (PFS) (B) according to the stage of primary colorectal diffuse large B‐cell lymphoma (DLBCL). Comparison of OS (C) and PFS (D) by stage between colorectal and gastric DLBCL. Comparison of OS (E) and PFS (F) between colorectal and gastric DLBCL based on prognostic index. Comparison of OS (G) and PFS (H) based on primary lymphoma resection status.

Ten patients (40.0%) underwent resection of their primary lymphoma at diagnosis, all of whom had limited‐stage disease. Among those patients, three underwent R1 resection with microscopic tumor involvement at the margins despite no visible residual disease. A CR rate of 80% (*n* = 20) was achieved in patients with primary colorectal DLBCL who underwent front‐line R‐CHOP therapy. The median OS and PFS for primary colorectal DLBCL were not reached, with estimated 3‐year rates of 86.7% and 72.3%, respectively, which were somewhat lower than in the overall cohort. Among 25 patients, two received consolidative RT, one of whom experienced relapse. In survival analysis, patients with limited‐stage disease demonstrated significantly better outcomes (Figure 4A,B). Notably, limited‐stage disease had a greater prognostic impact in primary colorectal DLBCL than in primary gastric DLBCL; however, the R‐IPI score was less meaningful (Figure 4C–F; Table 3). Although primary lymphoma resection was associated with better disease control, it did not lead to improved OS (Figure 4G,H).

**TABLE 3:** Univariate and multivariate analysis for overall survival and progression‐free survival of primary colorectal DLBCL.

Variables	HR (95% CI)	*p*
Univariate		
Primary lymphoma resection	0.019 (0.000–243.654)	0.412
Age ≥ 60	1.088 (0.098–12.047)	0.945
Male	1.892 (0.172–20.879)	0.603
ECOG‐PS ≥ 2	2.710 (0.245–29.962)	0.416
Stage ≥ II‐2	**19.302 (0.044–99.882)**	**0.034**
Initial B symptom	5.360 (0.471–60.956)	0.176
Elevated LDH	3.477 (0.315–38.396)	0.309
R‐IPI ≥ 3	2.371 (0.214–26.247)	0.481
Bulky (≥ 7 cm) disease	0.815 (0.074–9.001)	0.868
Cell of origin: GCB type	1.240 (0.112–13.688)	0.861
Epstein–Barr Virus ISH positive	0.041 (0.000–163.311)	0.680
Multivariate		
Primary lymphoma resection	0.014 (0.000–19.447)	0.248
Age ≥ 60	0.308 (0.050–1.888)	0.203
Male	1.352 (0.225–8.108)	0.741
ECOG‐PS ≥ 2	1.044 (0.174–6.253)	0.963
Stage ≥ II‐2	**13.529 (1.500–122.001)**	**0.020**
Initial B symptom	3.378 (0.347–32.870)	0.294
Elevated LDH	3.102 (0.515–18.692)	0.217
R‐IPI ≥ 3	1.883 (0.314–11.304)	0.489
Bulky (≥ 7 cm) disease	7.237 (0.806–64.966)	0.077
Cell of origin: GCB type	4.391 (0.729–26.451)	0.106
Epstein–Barr Virus ISH positive	0.041 (0.000–755.591)	0.606

*Note:* The items that are statistically significant are highlighted in bold.

Abbreviations: ECOG‐PS, eastern cooperative oncology group performance status; GCB, germinal center B‐cell; HR, hazard ratio; ISH, in situ hybridization; LDH, lactate dehydrogenase; R‐IPI, revised international prognostic index.

Figure 3 shows the clinical course of all 25 patients with primary colorectal DLBCL. Four bowel perforations occurred before, during, or after treatment. Except for one patient (Patient 22) who died from perforation during therapy, these events did not affect survival and occurred near the primary site. During follow‐up, five patients (20.0%) experienced relapse, including three (12.0%) with primary refractory disease. Among relapsed patients, two remained disease‐free after recently receiving chimeric antigen receptor (CAR) T‐cell therapy. Three deaths (12.0%) were observed, with only one attributed to lymphoma (Patient 5).

## 4 Discussion

In this study, we conducted a retrospective analysis of primary GI lymphoma, with a particular focus on primary colorectal DLBCL. Research on primary colorectal DLBCL is scarce, with most of the available literature limited to abstracts or case reports. Through the analysis of 25 patients with primary colorectal DLBCL, we sought to characterize the clinical features, evaluate responses to the current standard of treatment, and compare survival outcomes with other types of primary GI lymphoma.

In this multicenter retrospective analysis, primary GI lymphoma most commonly originated in the stomach, which accounting for approximately 60% of cases, followed by the small bowel and colorectum. Consistent with previous studies, primary gastric DLBCL showed comparatively better survival outcomes than other GI tract origins [14, 15], and primary small bowel DLBCL had the poorest outcome (Figure 2C,D). A possible explanation for this finding is that a significant proportion of primary small bowel DLBCL cases were diagnosed at an advanced stage. In the Republic of Korea, the cost‐effectiveness and widespread availability of EGD and colonoscopy, along with overt symptoms such as obstruction or bleeding, facilitate early detection of primary gastric and colorectal DLBCL. In contrast, primary small bowel DLBCL often presents with nonspecific symptoms, leading to delayed diagnosis. Furthermore, the possibility of secondary involvement from systemic nodal DLBCL cannot be excluded, which may further contribute to its poorer prognosis compared with other primary GI lymphomas.

Primary gastric DLBCL is the most common subtype among primary GI lymphomas, with treatment strategies relatively well‐established through consensus. Favorable survival outcomes of primary gastric DLBCL have diminished the need for surgery at diagnosis; however, primary intestinal DLBCL exhibits more aggressive behavior [16] and is often accompanied by complications such as bleeding and obstruction, leading to more frequent application of surgical intervention. In addition, surgery can reduce disease burden by removing the primary tumor and potentially metastatic lymph nodes. Nevertheless, the role of primary tumor resection in primary colorectal DLBCL remains controversial [7, 16, 17] and should be approached with caution due to the risks of procedure‐related complications and postoperative sequelae. In the present study, primary tumor resection appeared to contribute to local disease control. Although a trend toward improved PFS was observed, this finding should be interpreted with caution in light of the study's limitations, including its retrospective design, small cohort size, and potential selection bias. Surgical intervention was predominantly undertaken in patients with limited‐stage disease. Therefore, causal interpretation of its association with survival is constrained. In the limited‐stage subgroup, the absence of mortality precluded a definitive OS comparison by resection status; however, a trend toward improved PFS was observed (*p* = 0.083). These findings suggest that primary tumor resection may be considered for local control as part of rituximab‐containing chemoimmunotherapy in selected localized cases. Notably, even when the primary tumor was resected without achieving a sufficient negative margin (R1 resection), local disease control appeared to be maintained (Figure 4G,H). Therefore, while R0 resection is preferable when surgery is deemed necessary, aggressive procedures undertaken solely to secure negative margins may not be warranted.

In most lymphomas, limited‐stage disease is generally associated with favorable survival outcomes. However, this association was more pronounced in primary colorectal DLBCL than in primary gastric DLBCL in our study (Table 3; Figure 4C,D; Figure S1; Table S1), although this finding should be interpreted with caution given the small sample size and retrospective design. In addition, univariate and multivariate analyses yielded wide or boundary confidence intervals for several covariates, reflecting sparse‐event constraints. Notably, the conventional prognostic index for DLBCL, R‐IPI, was not a significant determinant of survival in primary colorectal DLBCL (Figure 4E,F). This discrepancy may arise from differences in staging methodology, specifically the use of the Lugano staging system instead of the Ann Arbor staging system in this study. However, a consensus on the optimal staging system for assessing primary GI lymphoma has yet to be reached. In one study, the T stage of the Paris classification system was suggested to possibly serve as a prognostic indicator [18], emphasizing the need to redefine risk stratification models to account for the unique characteristics of the disease.

Although relapsed or refractory primary GI lymphoma showed better survival outcomes than conventional relapsed nodal DLBCL, as reported in previous studies [19], the retrospective design and relatively small sample size in our study limit broader applicability. Selection bias may have influenced the decision to undergo surgery, and the lack of a standardized treatment protocol could have introduced detection bias. However, our study provides clinically meaningful real‐world data. Given the challenges in managing relapsed or refractory cases, the efficacy of novel therapies is of particular interest. Notably, two patients treated with CAR T‐cell therapy remained disease‐free, highlighting the potential of this approach in selected patients. Further research is warranted to clarify the role of novel cellular therapies in relapsed or refractory primary GI DLBCL.

In this study, we demonstrated the efficacy of front‐line R‐CHOP therapy in primary colorectal DLBCL. When clinically appropriate, surgical intervention may be considered in selected patients to achieve local control, but excessive resection aimed at securing negative margins should be avoided to prevent unnecessary tissue removal. In addition, traditional prognostic models developed for nodal DLBCL may not fully capture the behavior of primary GI DLBCL and should be refined to reflect its unique characteristics. Although survival outcomes in primary colorectal DLBCL were favorable, optimal management strategies for relapsed or refractory disease remain undefined. Continued research is necessary to address these challenges and improve outcomes in this rare subtype of lymphoma.

## Author Contributions

**Yong‐Pyo Lee:** conceptualization, investigation, methodology, data curation, writing – original draft, writing – review and editing, formal analysis, visualization. **Myung‐Won Lee:** conceptualization, investigation, methodology, data curation, writing – review and editing. **Seonggyu Byeon:** conceptualization, investigation, methodology, data curation, writing – review and editing.

## Ethics Statement

The institutional review boards of the participating hospitals (Chungnam National University Hospital, Chungbuk National University Hospital, and Gachon University Gil Medical Center) approved this study, and written informed consent was waived.

## Conflicts of Interest

The authors declare no conflicts of interest.

## Supporting information


**Figure S1:** Overall survival (A and B) and progression‐free survival (C and D) stratified by stage in primary colorectal and gastric diffuse large B‐cell lymphoma.

Table S1.

## Data Availability

For original data, please contact bichon3@cmcnu.or.kr or bichon3@catholic.ac.kr.
