# Supplementary figures and images for: Clinical Features and Outcomes of Primary Colorectal Diffuse Large B‐Cell Lymphoma: A Multicenter Retrospective Study
Source: Cancer Med. 2025 Oct 21;14(20):e71313. doi: 10.1002/cam4.71313 (PMC12538638; doi:10.1002/cam4.71313)

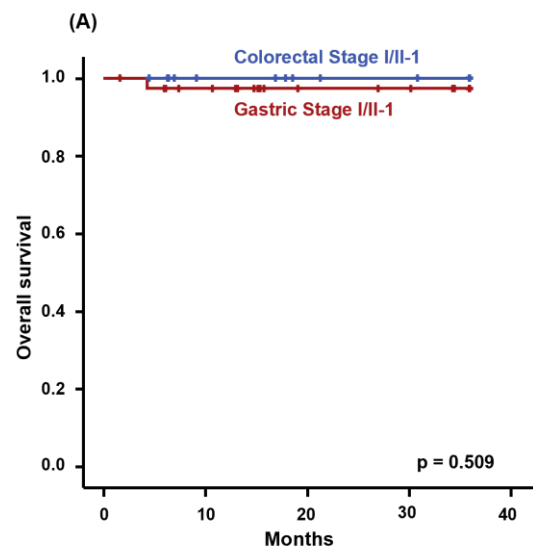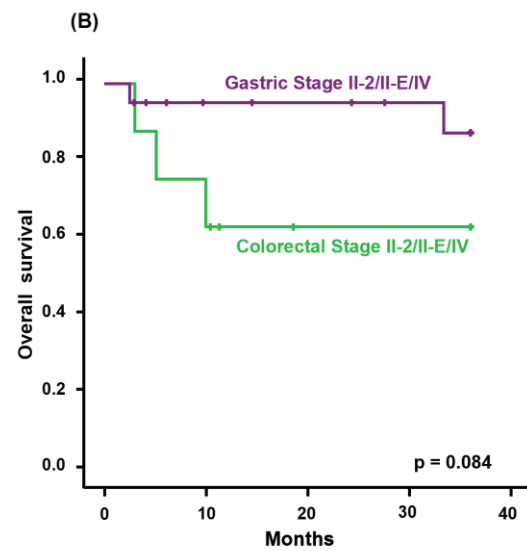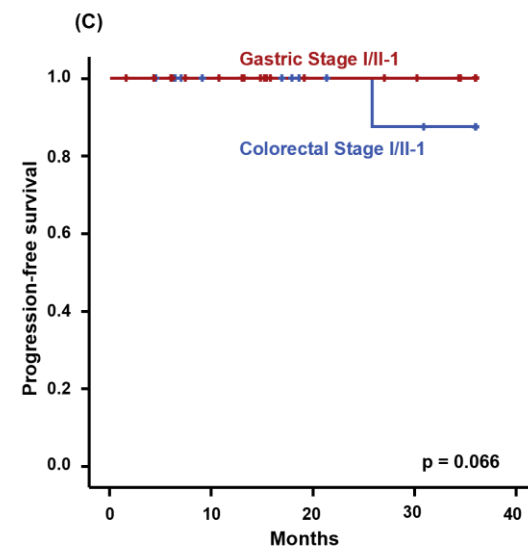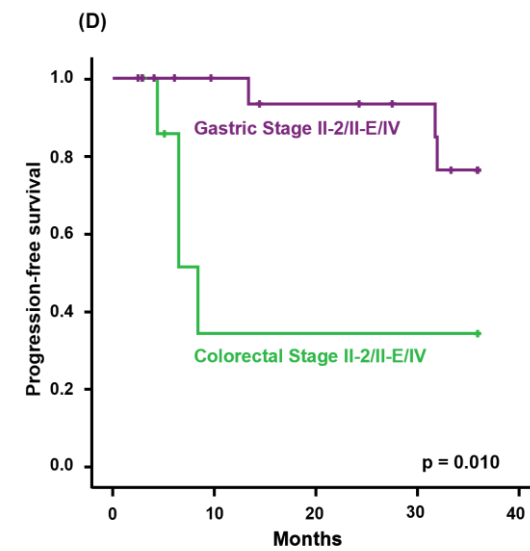

Supplement: Supplementary file 1 — Figure S1: Overall survival (A and B) and progression‐free survival (C and D) stratified by stage in primary colorectal and gastric diffuse large B‐cell lymphoma. [file CAM4-14-e71313-s001.pdf]
