# Supplementary material for: Clinical Features and Outcomes of Primary Colorectal Diffuse Large B‐Cell Lymphoma: A Multicenter Retrospective Study
Source: Cancer Med. 2025 Oct 21;14(20):e71313. doi: 10.1002/cam4.71313 (PMC12538638; doi:10.1002/cam4.71313)
Supplement: Supplementary file 2 — Table S1. [file CAM4-14-e71313-s002.docx]

**Supplementary Table 1.** Univariate and multivariate analysis for overall survival and progression-free survival of primary gastrointestinal DLBCL

| Variables | Univariate | | Multivariate | |
| --- | --- | --- | --- | --- |
|  | **HR (95%CI)** | ***p*** | **HR (95%CI)** | ***p*** |
| Age ≥ 60 | 7.341 (0.929 – 58.019) | 0.059 | 5.396 (0.614 – 47.454) | 0.129 |
| Male | 1.358 (0.383 – 4.816) | 0.635 | 1.525 (0.403 – 5.780) | 0.535 |
| ECOG-PS ≥ 2 | 8.561 (1.809 – 40.5517) | 0.007 | 6.139 (0.780 – 48.296) | 0.085 |
| Stage ≥ II-2 | 7.909 (1.677 – 37.306) | 0.009 | 2.612 (0.383 – 17.821) | 0.327 |
| Initial B symptom | 4.997 (1.255 – 19.889) | 0.022 | 0.933 (0.187 – 4.647) | 0.933 |
| Elevated LDH | **7.684 (1.629 – 36.257)** | **0.010** | **9.498 (1.329 – 67.855)** | **0.025** |
| R-IPI ≥ 3 | 9.651 (2.042 – 45.608) | 0.004 | 0.641 (0.061 – 6.719) | 0.710 |
| Cell of origin: GCB type | 0.504 (0.107 – 2.372) | 0.386 | 1.714 (0.290 – 10.134) | 0.552 |
| Epstein-Barr Virus ISH positive | 0.042 (0.000 – 321.254) | 0.487 | 0.000 (0.000 – N/A) | 0.987 |

**HR,** hazard ratio; **ECOG-PS**, eastern cooperative oncology group performance status; **LDH**, lactate dehydrogenase; **R-IPI**, revised international prognostic index; **GCB**, germinal center B-cell; **ISH**, in situ hybridization; **N/A**, non available

| Variables | Univariate | | Multivariate | | |
| --- | --- | --- | --- | --- | --- |
|  | **HR (95%CI)** | ***p*** | **HR (95%CI)** | | ***p*** |
| Age ≥ 60 | 1.625 (0.544 – 4.853) | 0.385 | 2.231 (0.596 – 8.356) | | 0.234 |
| Male | 0.647 (0.224 – 1.866) | 0.421 | 0.677 (0.224 – 2.051) | | 0.491 |
| ECOG-PS ≥ 2 | 1.867 (0.646 – 5.395) | 0.249 | 1.221 (0.237 – 6.291) | | 0.811 |
| Stage ≥ II-2 | 14.760 (3.296 – 66.088) | <0.001 | **15.443 (2.519 – 94.671)** | | **0.003** |
| Initial B symptom | 5.000 (1.349 – 18.541) | 0.016 | 3.148 (0.559 – 17.738) | 0.194 | |
| Elevated LDH | 3.010 (1.042 – 8.696) | 0.042 | 1.384 (0.333 – 5.749) | 0.654 | |
| R-IPI ≥ 3 | 5.303 (1.772 – 15.872) | 0.003 | 0.842 (0.186 – 3.823) | 0.824 | |
| Cell of origin: GCB type | 1.601 (0.555 – 4.619) | 0.384 | **3.928 (1.027 – 15.015)** | **0.046** | |
| Epstein-Barr Virus ISH positive | 0.042 (0.000 – 67.069) | 0.399 | 0.000 (0.000 – N/A) | 0.986 | |

**HR,** hazard ratio; **ECOG-PS**, eastern cooperative oncology group performance status; **LDH**, lactate dehydrogenase; **R-IPI**, revised international prognostic index; **GCB**, germinal center B-cell; **ISH**, in situ hybridization; **N/A**, non available
